# Chemical Basis of Floral Color Signals in Gesneriaceae: The Effect of Alternative Anthocyanin Pathways

**DOI:** 10.3389/fpls.2020.604389

**Published:** 2020-12-14

**Authors:** Ezgi Ogutcen, Karine Durand, Marina Wolowski, Laura Clavijo, Catherine Graham, Gaétan Glauser, Mathieu Perret

**Affiliations:** ^1^Conservatoire et Jardin botaniques de la Ville de Genève, Department of Botany and Plant Biology, University of Geneva, Geneva, Switzerland; ^2^Institute of Natural Sciences, Federal University of Alfenas, Alfenas, Brazil; ^3^Instituto de Ciencias Naturales, National University of Colombia, UNAL, Bogotá, Colombia; ^4^Swiss Federal Research Institute (WSL), Birmensdorf, Switzerland; ^5^Neuchatel Platform of Analytical Chemistry, University of Neuchatel, Neuchâtel, Switzerland

**Keywords:** Gesneriaceae, anthocyanin pathway, deoxyanthocyanin, visual systems, hummingbird pollination, floral pigments, chromatic signal

## Abstract

Changes in floral pigmentation can have dramatic effects on angiosperm evolution by making flowers either attractive or inconspicuous to different pollinator groups. Flower color largely depends on the type and abundance of pigments produced in the petals, but it is still unclear whether similar color signals rely on same biosynthetic pathways and to which extent the activation of certain pathways influences the course of floral color evolution. To address these questions, we investigated the physical and chemical aspects of floral color in the Neotropical Gesnerioideae (ca. 1,200 spp.), in which two types of anthocyanins, hydroxyanthocyanins, and deoxyanthocyanins, have been recorded as floral pigments. Using spectrophotometry, we measured flower reflectance for over 150 species representing different clades and pollination syndromes. We analyzed these reflectance data to estimate how the Gesnerioideae flowers are perceived by bees and hummingbirds using the visual system models of these pollinators. Floral anthocyanins were further identified using high performance liquid chromatography coupled to mass spectrometry. We found that orange/red floral colors in Gesnerioideae are produced either by deoxyanthocyanins (e.g., apigenidin, luteolinidin) or hydroxyanthocyanins (e.g., pelargonidin). The presence of deoxyanthocyanins in several lineages suggests that the activation of the deoxyanthocyanin pathway has evolved multiple times in the Gesnerioideae. The hydroxyanthocyanin-producing flowers span a wide range of colors, which enables them to be discriminated by hummingbirds or bees. By contrast, color diversity among the deoxyanthocyanin-producing species is lower and mainly represented at longer wavelengths, which is in line with the hue discrimination optima for hummingbirds. These results indicate that Gesnerioideae have evolved two different biochemical mechanisms to generate orange/red flowers, which is associated with hummingbird pollination. Our findings also suggest that the activation of the deoxyanthocyanin pathway has restricted flower color diversification to orange/red hues, supporting the potential constraining role of this alternative biosynthetic pathway on the evolutionary outcome of phenotypical and ecological diversification.

## Introduction

Flower color plays a key role in angiosperm reproduction by attracting pollinators. Floral color acts as a visual cue for pollinators to associate certain visual signals with reward (Chittka and Menzel, 1992; Schiestl and Johnson, 2013). Not all pollinators have the same color vision, and different pollinator groups perceive and select flowers differently. Bees have trichromatic vision with photoreceptors sensitive to UV, blue, and green, which enables them to detect short- to medium-wavelengths (Chittka, 1992). On the other hand, flower-visiting birds, such as hummingbirds, have a tetrachromatic color vision with photoreceptors sensitive to UV, blue, green, and red, which enables them to detect not only short- and medium-, but also long-wavelengths (Herrera et al., 2008; Stoddard et al., 2020). Adaptations to these differences in pollinator visual processing have been considered as a major driver of floral color diversification in angiosperms and the repeated evolution of color signals specific to certain functional pollinator groups. For instance, bee-pollinated flowers tend to have reflectance between 400 and 500 nm wavelengths, which matches the discrimination optima for bees (von Helversen, 1972; Dyer et al., 2012). On the other hand, bird-pollinated flowers frequently have a distinctive orange/red color, which is better detected by birds than by bees especially against a green vegetation background (Lunau et al., 2011; Shrestha et al., 2013; Burd et al., 2014; Bergamo et al., 2016). Despite the importance of color signaling in plant-pollinator interactions, the biochemical basis of color shifts has been investigated in a limited number of studies (Hoballah et al., 2007; Sobel and Streisfeld, 2013; Ng and Smith, 2016), and our understanding of how pigment biosynthesis pathways shaped the evolution of floral color is still limited (Cronk and Ojeda, 2008; Rausher, 2008; Ng et al., 2018; Wheeler and Smith, 2019).

**FIGURE 1:**
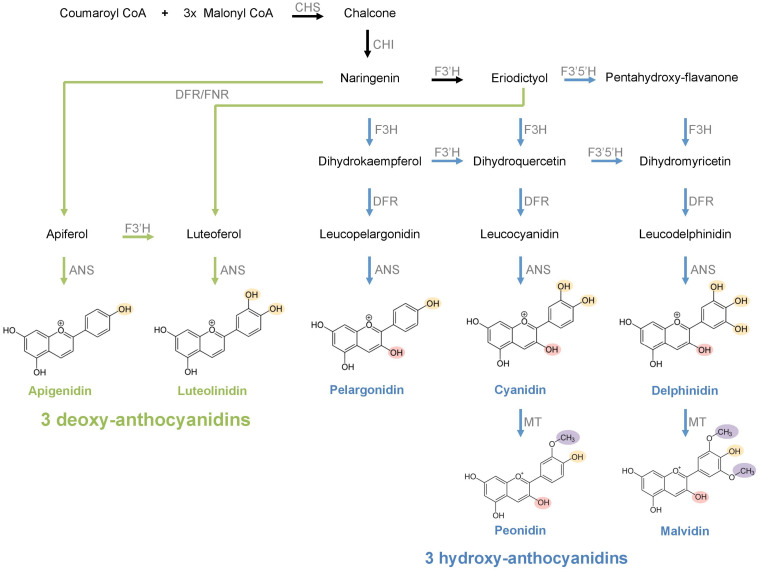
The anthocyanin biosynthesis pathway. The reactions leading to hydroxy- and deoxy-anthocyanin compounds are represented in blue and green arrows, respectively. Earlier reactions preceding the two alternative pathways are represented in black arrows. OH groups differing between hydroxy- and deoxy-anthocyanins are colored in pink. OH groups in varying numbers in different anthocyanidins are colored in yellow. CH_3_ groups in different hydroxy-anthocyanins are colored in purple. CHS, chalcone synthase; CHI, chalcone isomerase; F3′H, flavonoid 3′-hydroxylase; F3′5′H, flavonoid 3′,5′-hydroxylase; DFR, dihydroflavonol 4-reductase; FNR, flavone synthase; ANS, anthocyanidin synthase; MT, methyltransferase.

As one of the most common floral pigment groups, anthocyanins are water-soluble flavonoid compounds that produce a broad range of hues including blue, purple, pink, orange, and red (Tanaka et al., 2008; Zhao and Tao, 2015). The anthocyanin biosynthesis pathway (ABP; Figure 1) involves several structural and regulatory proteins that orchestrate the production of a wide array of pigments derived from three 3-hydroxylated anthocyanidin precursors: pelargonidin, cyanidin, and delphinidin (Holton and Cornish, 1995; Albert et al., 2014). In addition to these 3-hydroxyanthocyanins, the ABP can also produce 3-deoxyanthocyanins (e.g., apigenidin, luteolinidin), which lack the hydroxyl group on carbon 3 and are derived from an alternative branch of the ABP that uses flavanone as a substrate (Winefield et al., 2005; Grotewold, 2006; Piatkowski et al., 2020). Unlike 3-hydroxyanthocyanins, 3-deoxyanthocyanins produce limited hues ranging from orange to red and they are much less common in plants. These uncommon anthocyanin pigments have only been detected in ferns, mosses, few members of Poaceae (in sorghum, maize, and sugarcane) and Theaceae (tea), and some New World clades of Bignoniaceae and Gesneriaceae so far (Harborne, 1966, 1967; Winefield et al., 2005; Piatkowski et al., 2020). While the biochemical production of floral anthocyanins has been extensively studied, the relative importance of deoxyanthocyanins in floral color and its detection by pollinators still needs to be evaluated.

Gesnerioideae is the Neotropical subfamily of Gesneriaceae, and it consists of 75 genera with over 1,200 species (Perret et al., 2013). The outstanding floral color diversity, the presence of both hydroxy- and deoxy-anthocyanins pigments, and several transitions between hummingbird and bee pollination syndromes throughout the evolution of this clade make Gesnerioideae an appropriate study system to examine the biochemical mechanisms underlying floral color transitions and their impact on plant-pollinator interactions (Roberts and Roalson, 2017, 2020; Serrano-Serrano et al., 2017). Based on the presence of two alternative branches of the ABP in the Gesnerioideae, we predict that (i) the deoxyanthocyanin-producing plants have an additional biochemical solution to produce orange/red flowers, and that (ii) these plants are limited to producing orange/red hues, which results in reduced floral color diversity and a higher degree of specialization on hummingbird pollination when compared to the hydroxyanthocyanin-producing lineages. In this study, we aim to test these predictions by evaluating the relative contributions of the hydroxyanthocyanin and deoxyanthocyanin branches of the ABP to the evolution of orange/red flowers in the Gesnerioideae. To achieve this, we identified the anthocyanin compositions and measured reflectance spectra of hummingbird- and bee-pollinated flowers representing 156 species from different Gesnerioideae lineages. We also used hummingbird and bee visual models to evaluate how anthocyanin composition affects flower detection by pollinators. Our results will contribute to a better understanding of the chemical basis of color signals and how alternative branches of the ABP may affect floral color diversification.

## Materials and Methods

### Sampling

We collected pigment and reflectance data from 180 samples representing 156 Gesnerioideae species, exhibiting the full range of floral color diversity and pollination syndromes within this Neotropical clade (Supplementary Table S1). Flowers were collected from plants cultivated at the Botanical Garden of Geneva (Switzerland), in Mauro Peixoto private collection (Sao Paulo, Brazil), and during field trips in Brazil, Colombia, and Ecuador (see Supplementary Table S1 for voucher information). Observation-based pollinator information was available for 56 species (see Supplementary Table S2 for references).

### Detection and Identification of Anthocyanin Pigments

Anthocyanin pigments were extracted from fresh or silica-dried corollas using methanol:HCl (99.9:0.1, v/v). Anthocyanin profiling was performed by ultra-high performance liquid chromatography-diode array detection-quadrupole time-of-flight mass spectrometry (UHPLC-DAD-QTOFMS). The UHPLC system was an Acquity UPLC (Waters, Milford, MA), the DAD detector an eλ PDA (Waters), and the QTOFMS a Synapt G2 (Waters). The entire system was controlled by Masslynx 4.1 (Waters). One microliter of extract was injected on an Acquity UPLC CSH C18 column (100 × 2.1 mm i.d., 1.7 μm particle size) maintained at 45°C. Mobile phases consisted of H_2_O supplemented with formic acid 0.15% (phase A) and acetonitrile supplemented with formic acid 0.05% (phase B). The flow rate was of 0.4 mL/min and the following gradient was applied: 2–30% phase B in 6 min, 30–100% phase B in 2 min, hold at 100% phase B for 2 min, and re-equilibration at 2% phase B for 4 min. The DAD parameters were set as follows: range 190–600 nm, resolution 1.2 nm, sampling rate 20 Hz, time constant 0.1 s. The mass spectrometer was operated in electrospray positive ionization over a mass range of 85–1,200 Da. The so-called MSe mode was used, in which two acquisition functions are set in parallel: a low fragmentation (collision energy 4 eV) function yielding ions of the molecular species, and a high fragmentation (ramp 10–30 eV) function yielding fragments of interest. Accurate mass measurements were obtained by infusing a solution of the synthetic peptide leucine-enkephalin (m/z 556.2771 Da) in the mass spectrometer. The detection and identification of anthocyanins were performed by (1) selecting all peaks absorbing between 480 and 530 nm in the DAD chromatogram and reporting absorbance spectra and maxima; (2) extracting the corresponding peaks in the low fragmentation mass spectrum and computing the most probable molecular formulae; and (3) interpreting the aglycone and the sequence of sugars from the high fragmentation mass spectrum. The Dictionary of Natural Products^1^ was further used to identify the detected anthocyanins. These anthocyanins were integrated in the software TargetLynx^TM^ (Waters) and quantified using calibration curves built from standard solutions of luteolinidin, apigenidin and pelargonidin-3-rutinoside at 0.2, 1, 5, 20, and 100 μg/mL.

To simplify the chemistry data for downstream analyses, we divided the samples into four categories. “DEO90” and “HYD90” categories represent samples with more than 90% deoxyanthocyanin and hydroxyanthocyanin pigments, respectively. The third category is labeled as “DEO+HYD” and it includes samples that produce between 10 and 90% of both pigments. The fourth category includes samples with no anthocyanin.

A principal component analysis (PCA) biplot was generated using the R packages ade4 (Dray and Dufour, 2007) and factoextra (Kassambara and Mundt, 2020). The anthocyanin compositions were coded as continuous variables based on the relative percentages of the different anthocyanidins present in each sample. In order to account for the phylogenetic signal, phylogenetic PCA was also performed using two correlation structure methods (Brownian motion and Pagel’s lambda with maximum likelihood optimization) implemented in the R package phytools (Revell, 2012).

### Quantification of Floral Color

We quantified floral color on fresh corollas by measuring spectral reflectance for wavelengths between 300 and 700 nm using a portable JAZ spectrometer with a UV-VIS light source (Ocean Optics, Inc., Dunedin, FL). We calibrated the spectrometer using a Spectralon reflectance standard WS-1 (Ocean Optics, Inc., Dunedin, FL), and performed the measurements at a 45° angle with the probe shielding from any ambient light to avoid background noise. We measured one to three flowers per sample at up to three different points on the corollas. For flowers with uniform coloration or minor colored patterns (e.g., small spots or stripes), all measurements were averaged to obtain a single spectrum for each sample. For flowers with different lobe and tube colors, measurements for each part were averaged separately, resulting in two spectra per sample. We processed the reflectance data on the R package PAVO (Maia et al., 2013, 2019), and generated reflectance plots using LOESS smoothing with a smoothness parameter of 0.25.

We followed the method described by Chittka et al. (1994) to classify flower color. We divided the color spectrum into four regions: (i) 300–400 nm as UV; (ii) 400–500 nm as blue; (iii) 500–600 nm as green; and (iv) 600–700 nm as red. The region was coded “+” if the reflectance values within that region were above 10%, and coded “−” if below 10%. When the reflectance values above 10% were measured near the upper-boundary of the region, the region was coded as “/.” Based on this floral color classification, the UV-b-g-r+ and UV-b-g/r+ flowers were coded as “orange/red,” and the other flowers were coded as “other colors.”

### Ancestral State Reconstruction

The evolution of deoxyanthocyanin production was reconstructed on the Gesnerioideae phylogeny from Serrano-Serrano et al. (2017), after having pruned the tree to only include the 156 study species. Maximum likelihood-based ancestral reconstruction was performed using Markov-k state 1 model implemented in Mesquite 3.03 (Maddison and Maddison, 2014). The presence and absence of anthocyanin production were coded as 1 and 0, respectively. The percentage of deoxyanthocyanin present in each sample was mapped onto the Gesnerioideae phylogeny using the method anc.ML (Ancestral character estimation using likelihood) under the function *contMap* (Map continuous trait evolution on the tree) implemented the R package phytools (Revell, 2012). As outgroup, we selected Didymocarpoideae, which was previously shown to produce no deoxyanthocyanins (Harborne, 1966, 1967).

### Visual Models for Bees and Hummingbirds

In order to visualize how different pollinators perceive floral colors, we used standard color space models for bees and hummingbirds that are included in the *vismodel* function of the R package PAVO (Maia et al., 2013, 2019). For bee vision, we used the visual mode “*Apis mellifera*,” and we set the longest-wavelength photoreceptor as achromatic, and green foliage as background. We visualized the samples on the two-dimensional hexagonal color space that is used for trichromatic visual systems (Chittka, 1992). For hummingbird vision, we used the visual mode “*average avian V system*” and set green foliage as background. We visualized the samples on the three-dimensional tetrahedral color space that is used for tetrachromatic visual systems (Herrera et al., 2008).

For the two-dimensional bee visual space, we calculated the areas occupied by “DEO90” and “HYD90” samples using the function *mcp* (minimum convex polygon) implemented in the R package adehabitatHR (Calenge, 2006). In order to test the hypothesis that the DEO90 samples occupy a smaller area than a subset of samples randomly selected from the whole dataset, we performed 100 random subsampling and obtained confidence intervals for the area occupied by the random subsets. Random subsampling was performed using the RAND function on Microsoft Excel and the area calculations of the subsets were performed using the *mcp* function in the R package adehabitatHR (Calenge, 2006) as described above. Confidence intervals and the *p*-values were calculated using the R package gmodels (Warnes, 2005).

### Estimation of the Color Discrimination Ability of Pollinators

A color is best discriminated by a visual system if there is a rapid change in the reflectance values along the spectrum where the sensitivities of different photoreceptors overlap (Chittka and Menzel, 1992). To estimate how well the flowers are discriminated by bee and hummingbird visual systems, we quantified the location of these reflectance changes on the spectrum by calculating the middle point of a steep curve (marker points), where the change was higher than 20%, as previously described (Chittka and Menzel, 1992; Dyer et al., 2012; Shrestha et al., 2013). We used the function *peakshape* in the R package PAVO (Maia et al., 2013, 2019) to calculate the marker points for each sample. Depending on the shape of the reflectance curve, the total number of marker points per sample ranged from one to three (Supplementary Table S1).

We quantified the match between marker points and the color discrimination optima for bees and hummingbirds using two metrics described in Shrestha et al. (2013). Color discrimination optima for visual systems of bees (400 and 500 nm) and hummingbirds (460, 540, and 600 nm) were obtained from previous studies (von Helversen, 1972; Emmerton and Delius, 1980). The first metric used in this method is the mean absolute deviation (MAD), which is calculated as the average absolute difference between each marker point and the closest color discrimination optimum of a vision system. The second metric, minimum absolute deviation (minAD), considers each visual optimum separately and is obtained by calculating the absolute distance of the closest marker point to the visual optimum. For both MAD and minAD, small and large values represent high and low discrimination abilities, respectively.

We used phylogenetic ANOVA within the R package phytools (Revell, 2012) and the Gesnerioideae phylogeny (Serrano-Serrano et al., 2017) to test the differences in the MAD_Bee and MAD_Hummingbird values between the DEO-dominant (>50% deoxyanthocyanidin) and HYD-dominant (>50% hydroxyanthocyanidin) flowers. We performed 10,000 simulations and used Holm-Bonferroni method to adjust *p*-values for multiple comparisons.

## Results

### Anthocyanin Identification and Composition in the Gesnerioideae

According to HPLC retention times, UV-vis absorption spectral characteristics, and high-resolution MS/MS spectra, we identified 12 anthocyanins (nine hydroxyanthocyanins and three deoxyanthocyanins) from the 180 samples (Supplementary Tables S1, S3). The aglycone parts of these 12 compounds correspond to five hydroxyanthocyanidins (pelargonidin, cyanidin, peonidin, malvidin, and delphinidin), and three deoxyanthocyanidins (apigenidin, luteolinidin, and an unidentified deoxy-anthocyanin pigment). We detected no anthocyanins in 28 samples. In the remaining 152 samples, pelargonidin (in 84 samples), cyanidin (52), malvidin (40), and luteolinidin (39) were the four most commonly produced anthocyanins. The anthocyanin composition was variable among the samples: 35.6% of the samples had a single anthocyanidin type, whereas the remaining samples produce a mix of two (27.2%), three (13.9%), or four to six different anthocyanidins (7.8%). Some anthocyanidin combinations were more frequently observed than the others (Supplementary Figure S1). The three most common combinations were (i) pelargonidin and cyanidin/peonidin (in 36 samples); (ii) cyanidin/peonidin and delphinidin/malvidin (33); and (iii) pelargonidin and delphinidin/malvidin (27). Apigenidin was the least common anthocyanidin, and it was only observed in combination with other anthocyanidins.

**FIGURE 2:**
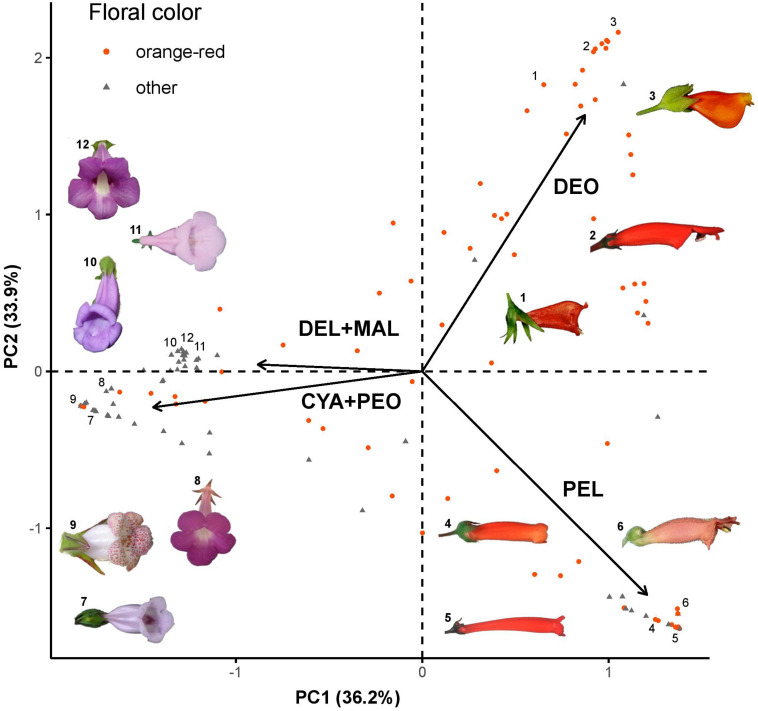
PCA plot of Gesnerioideae flowers based on their anthocyanin composition. Arrows represent 4 loadings: PEL, pelargonidin; CYA+PEO, cyanidin and peonidin; DEL+MAL, delphinidin and malvinidin; DEO, deoxyanthocyanin (luteolinidin and apigenidin). Orange/red flowers are shown as orange dots, flowers with other colors are shown as gray triangles. 1: *Nematanthus crassifolius*, 2: *Sinningia glaziovana*, 3: *N. strigillosus*, 4: *S. aggregata*, 5: *S. gigantifolia*, 6: *S. elatior*, 7: *Paliavana tenuiflora*, 8: *Achimenes grandiflora*, 9: *N. whieleri*, 10: *S. sp. nov.1*, 11: *S. eumorpha*, and 12: *S. speciosa*.

The PCA based on the relative frequencies of each anthocyanidin showed that sampled flowers are clustered according to their anthocyanidin composition (Figure 2). The first two principal components explained the 70.1% of the variation within the dataset, while the addition of the third principal component increased this percentage to 99.4% (Supplementary Figure S2). PC1 mainly reflected the abundance of the orange/red anthocyanidins (pelargonidin and the deoxyanthocyanins) versus other anthocyanidins. PC2 roughly corresponded to the presence of deoxyanthocyanins versus hydroxyanthocyanins. We observed that orange/red flowers were mostly clustered on right side of the plot, linked to the pelargonidin and deoxyanthocyanidin loadings, but we observed some orange/red flowers that were linked to the cyanidin/peonidin and delphinidin/malvinidin loadings. These results demonstrate that both hydroxy- and deoxy-anthocyanins contribute to the production of orange/red flowers in the Gesnerioideae. The results of the phylogenetic PCA were almost identical to the regular PCA under both Brownian motion and Pagel’s lambda models (λ = 0.000066), indicating that the effect of phylogenetic signal on the clustering of samples was negligible.

### The Evolution of Deoxyanthocyanin Production

**FIGURE 3:**
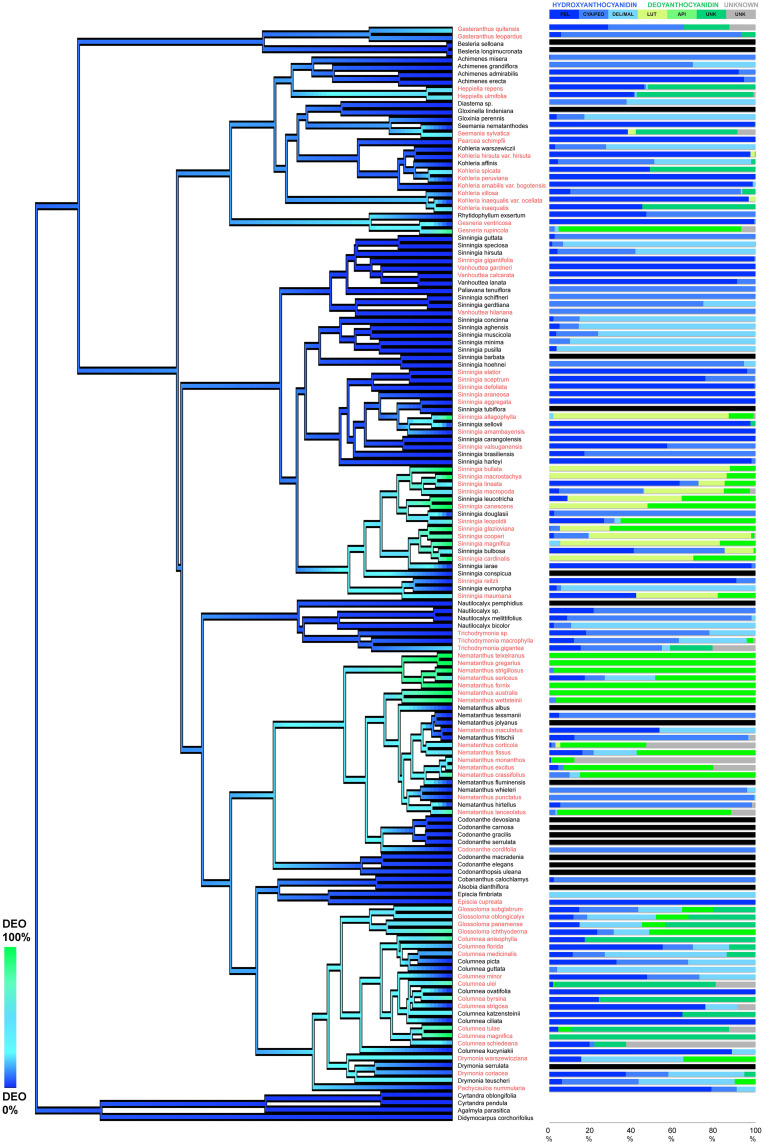
Evolution of deoxyanthocyanin production in the Gesnerioideae. The tree branches are colored based on the reconstructed percentage of the deoxy-anthocyanin presence in the flowers of each species. Species with orange/red flowers are highlighted in red. On the right side of the phylogeny, the percentages of each anthocyanidin are presented in color-coded bars. No-anthocyanin samples are labeled as black bars. PEL, pelargonidin; CYA/PEO, cyanidin and peonidin; DEL/MAL, delphinidin and malvinidin, API, apigenidin, LUT, luteolinidin; UNK (in green): unknown deoxyanthocyanidin; UNK (in gray): unknown anthocyanin. The phylogenetic tree derived from Serrano-Serrano et al. (2017).

Deoxyanthocyanins were identified in four subtribes and were particularly common in the genera *Columnea, Glossoloma, Nematanthus*, and *Sinningia* (Figure 3 and Supplementary Table S1). The di-hydroxylated luteolinidin-5-glucoside was detected dominantly in the genus *Nematanthus*, whereas the mono-hydroxylated apigenidin-5-glucoside was mainly restricted to the genus *Sinningia* suggesting that the regulation of deoxyanthocyanin production may differ among clades. The ancestral state reconstruction analysis showed that the activation of the deoxyanthocyanin pathway has evolved (and occasionally been lost) several times throughout the evolutionary history of the Gesnerioideae (Figure 3). These independent evolutions of deoxyanthocyanin production suggest convergent evolution of this anthocyanin type within the Gesnerioideae.

### The Color Diversity of the Gesnerioideae Flowers

**FIGURE 4:**
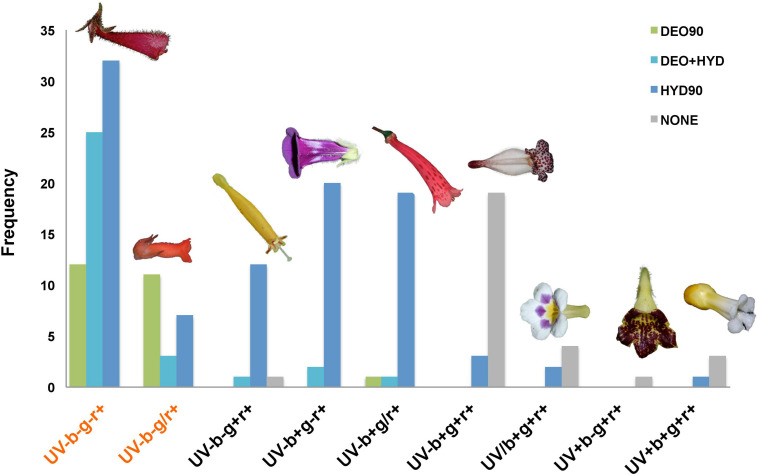
Color groups in the Gesnerioideae flowers. UV: UV (300–400 nm), b: blue (400–500 nm), g: green (500–600 nm), r: red (600–700 nm). –, /, + symbols represent no reflectance, partial reflectance, and full reflectance in the specified region of the spectrum. Color groups representing orange/red hues are shown in red text. DEO90 (green): samples with >90% DEO; DEO+HYD (cyan): samples with both DEO and HYD between 10 and 90%; HYD90 (blue): samples with >90% HYD; NONE: no-anthocyanin samples. Flowers representing each color group are shown at the top of each category. From left to right: *Nematanthus monanthos, Sinningia allagophylla, Columnea purpureamarginata, S. bragae, S. douglasii, N. punctatus, Diastema* sp., *Gasteranthus leopardus*, and *Besleria selloana*.

Based on the previously established floral color classification (Chittka et al., 1994), we observed 9 different color groups within the Gesnerioideae (Figure 4). The UV-b-g-r+ and UV-b-g/r+ groups were coded as orange/red flowers and they constitute 50% (90 out of 180) of the studied samples. 25.6% of the orange/red flowers were DEO90, 31.1% were DEO+HYD, and 43.3% was HYD90. The rest of the color groups, which included white, cream, yellow, purple, pink, and green flowers, the majority of them were HYD90 (63.3%), 31.1% had no anthocyanins, and very few were DEO90 (1.1%) or DEO+HYD (4.4%). These non-orange/red DEO90 and DEO+HYD flowers have pink or brown hues, which may seem orange/red to human eye, but they are not categorized as “orange/red” according to our color classification.

**FIGURE 5:**
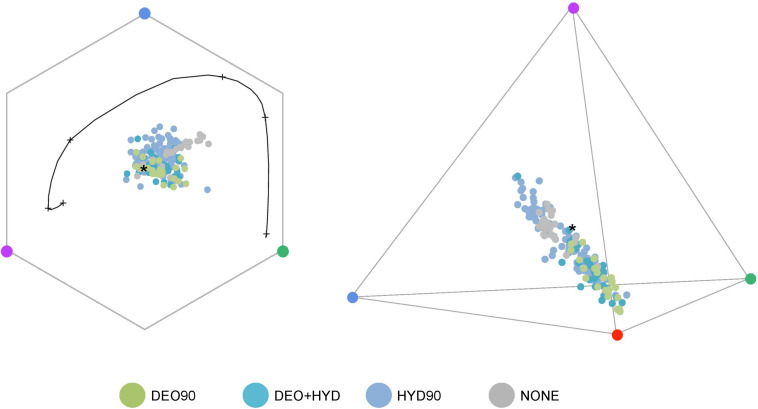
The color loci of Gesnerioideae flowers plotted in bee and hummingbird visual systems. HYD90 (>90% HYD; blue), DEO90 (>90% DEO; green), DEO+HYD (both DEO and HYD between 10 and 90%; cyan) and no-anthocyanin (NONE; gray) samples are shown in the hexagonal color-space of the bee visual system (left) and the tetrahedral color-space of the hummingbird visual system (right). Colored circles in each corner represents a photoreceptor: UV (purple), blue (blue), green (green), and red (red). * represents the achromatic center of the color-space. The black curve on the hexagonal color-space represents the maximum sensitivity of each photoreceptor to a monochromatic light.

We observed that the HYD90 samples occupy a larger space than the DEO90 samples in both hexagonal (bee) and tetrahedral (hummingbird) color spaces (Figure 5). Subsampling analysis of 100 random subsets suggests that the space occupied by the DEO90 samples is significantly smaller than any space occupied by random subsets (*p* < 0.0001).

We observed some intraspecific variation in our dataset. In many cases, species with multiple samples were highly similar in terms of both floral color and pigment composition (e.g., *Drymonia serrulata, Sinningia speciosa*). However, few species with multiple samples showed some variation in pigment compositions (e.g., *Glossoloma ichthyoderma*, *Nematanthus crassifolius*), or floral color (e.g., *Kohleria spicata*, *Nematanthus fornix*).

### Effect of the Anthocyanin Type on Floral Color Discrimination by Pollinators

**FIGURE 6:**
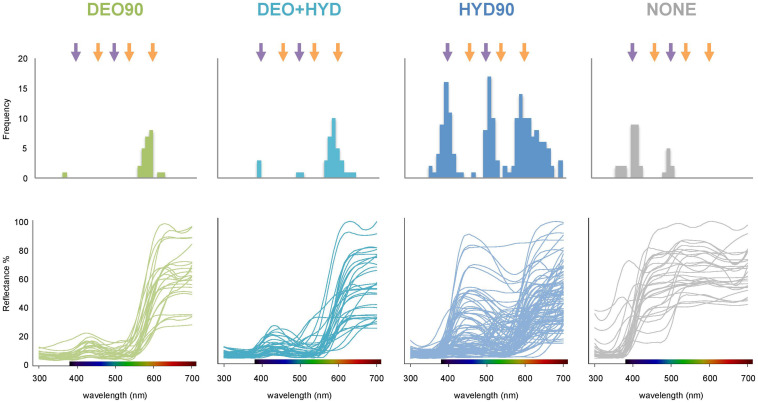
Marker point distribution (top panel) and the reflectance curves (bottom panel) of HYD90 (>90% HYD; blue), DEO90 (>90% DEO; green), DEO+HYD (both DEO and HYD between 10 and 90%; cyan) and no-anthocyanin (NONE; gray) flowers. Purple arrows at 400 and 500 nm represent the hue-discrimination optima for bees, and orange arrows at 460, 540, and 600 nm represent the hue-discrimination optima for hummingbirds. The color panels on the x-axes of the reflectance plots represent the visible light spectrum.

To investigate the visual capacities of bees and hummingbirds, we compared the difference in the marker point distribution among the samples (Figure 6). We found that marker points for the DEO90 and DEO+HYD samples concentrated around 600 nm, where the long-wavelength sensitivity of hummingbird vision is highest. However, the marker points for the HYD90 samples were concentrated not only around 600 nm, but also around 400 and 500 nm, where the short- and medium-length wavelength sensitivity of bee-vision is highest. In other words, whereas DEO90 and DEO+HYD flowers better match the hummingbird visual system, HYD90 flowers match the visual systems of both pollinators.

Concordant with the marker point distribution patterns, the values for MAD_Bee (*p* = 0.001) and MAD_bird (*p* = 0.0001) were significantly lower in HYD-dominant and DEO-dominant samples, respectively (Table 1 and Supplementary Table S1). Lower MAD values indicate better fit for the visual system, meaning that the HYD-dominant samples were better discriminated by bees, whereas DEO-dominant samples were better discriminated by hummingbirds. For minAD values, the most significant differences between the two groups were at the short-wavelength end of the spectrum. When compared to the DEO-dominant samples, HYD-dominant samples had significantly lower minAD400 (short-wavelength receptor for bee vision; *p* = 0.0001) and minAD460 (short-wavelength receptor for hummingbird vision; *p* = 0.0001) values, indicating that HYD-dominant samples were better discriminated by the short-wavelength receptors for both bee and hummingbird visual systems. Lastly, minAD600 values were lower in the DEO-dominant samples than the HYD-dominant samples, which indicates that DEO-dominant samples were better discriminated by the long-wavelength receptors of the hummingbird visual system.

**TABLE 1:** Statistical summary of MAD and minAD values for bee and hummingbird visual systems and their comparison between HYD-dominant (>50% HYD) and DEO-dominant (>50% DEO) samples.

		HYD-dominant (*n* = 111)	DEO-dominant (*n* = 41)	*p*-value (phylANOVA)
MAD_Bee	Mean	69.10	83.20	0.001*
	SD	33.78	13.44	
	SEM	3.21	2.10	
MAD_Bird	Mean	29.41	17.07	0.0001*
	SD	17.38	9.84	
	SEM	1.65	1.54	
minAD400	Mean	101.93	176.61	0.0001*
	SD	89.45	38.67	
	SEM	8.49	6.04	
minAD500	Mean	64.35	85.90	0.0981
	SD	43.95	11.24	
	SEM	4.17	1.76	
minAD460	Mean	88.61	122.46	0.0001*
	SD	45.54	15.71	
	SEM	4.32	2.45	
minAD540	Mean	49.87	44.90	0.051
	SD	27.47	11.24	
	SEM	2.61	1.76	
minAD600	Mean	34.14	16.51	0.0029*
	SD	36.71	8.97	
	SEM	3.48	1.40	

Lower MAD and minAD values indicate higher discrimination abilities. *Indicates statistical significance. SD, standard deviation; SEM, Standard error of the mean.

According to the observed pollinator data, out of 52 hummingbird-pollinated flowers, 10 are DEO90, 18 are DEO+HYD, 21 are HYD90, and 3 have no anthocyanins. Out of 16 bee-pollinated flowers, 9 are HYD90, 1 is DEO+HYD, and 6 have no anthocyanins. In other words, all DEO90 and all but one DEO+HYD flowers are exclusively pollinated by hummingbirds (28 samples), whereas HYD90 samples are pollinated by either bees (9 samples) or hummingbirds (21 samples).

## Discussion

Changes in floral color have direct impact on the attraction of different pollinator groups, which in turn has a significant effect on angiosperm evolution. However, the biochemical basis of flower color and its impact on flower diversity are only partially understood (Cronk and Ojeda, 2008; Rausher, 2008). Using chemical analysis of anthocyanin pigments and spectral reflectance measurements of 156 species, we found that floral color in the Neotropical Gesneriaceae relies on the biosynthesis of 12 different compounds including not only the common hydroxyanthocyanins but also the rare deoxyanthocyanins that have evolved independently in several lineages. Whereas the hydroxyanthocyanin-producing flowers display a wide range of colors, the deoxyanthocyanin-producing flowers show limited orange/red hues that are discriminated better by hummingbirds than by bees. We also found that the deoxyanthocyanin-producing species were more frequently pollinated by hummingbirds than the hydroxyanthocyanin-producing ones, suggesting that this alternative biochemical pathway may not only constrain the course of floral color evolution but also limit the number of suitable types of pollinators.

### The Alternative Branches of the Anthocyanin Pathway in the Gesnerioideae

In most angiosperms that use the ABP, floral color is produced by the common hydroxyanthocyanin derivatives, but the Neotropical Gesneriaceae is one of the few known clades that produces 3-deoxyanthocyanins via an alternative branch of the ABP. We detected these uncommon anthocyanin pigments in more than 30% of the flowers sampled, either alone or in combination with hydroxyanthocyanins. Our result considerably extend the number of Gesnerioideae species with deoxyanthocyanins, which was first documented in the pioneer work of Harborne (1966, 1967) who stressed the potential taxonomical utility of these pigments as a diagnostic feature of the Gesnerioideae subfamily.

To date, deoxyanthocyanins have been mainly recorded in the vegetative parts of disparate groups of seedless plants (mosses and ferns) and angiosperms, where they play a role in defense response against microbial infection and environmental stress (Kawahigashi et al., 2016; Xiong et al., 2019; Piatkowski et al., 2020). Within angiosperms, deoxyanthocyanins have been identified in the leaves of important crops like maize, sorghum and sugarcane (Poaceae), in black tea (*Camellia sinensis*, Theaceae) and in Bignoniaceae (e.g., *Fridericia chica).* Similar to these plant groups, the Gesnerioideae also produce deoxyanthocyanins in their leaves (Harborne, 1967). However, deoxyanthocyanin production in floral tissue and its contribution to pollination signaling is unique to this subfamily.

The frequent presence of both deoxy- and hydroxy-anthocyanins in the same flower indicates that the utility of these two alternative branches of the ABP are not mutually exclusive. We inferred several gains and losses of deoxyanthocyanin production throughout the evolution of the Gesnerioideae, which suggests that the shifts between the two alternative branches of the ABP were frequent and reversible in this subfamily (Figure 3). At the molecular level, dihydroflavonol 4-reductase (DFR) and flavanone-3-hydroxylase (F3H) are the key genes for the transition between the hydroxy- and the deoxy-anthocyanin branches of the ABP. In *Sinningia cardinalis*, upregulation of DFR and downregulation of F3H were linked to deoxyanthocyanin production (Winefield et al., 2005). This is achieved by generating flavanone in excess instead of dihydroflavonols, which are subsequently reduced to form the flavan-4-ols precursors rather than the usual flavan-3,4-diol precursors (Stich and Forkmann, 1988a,b). Therefore, the relative proportion of deoxy- and hydroxy-anthocyanins in the Gesnerioideae flowers could be potentially explained by the differences in the regulation of the DFR and F3H, and by different substrate specificity of the DFR copies, which was previously shown in other plant groups (des Marais and Rausher, 2008).

In addition to deoxyanthocyanidins, several hydroxyanthocyanidins (pelargonidin, cyanidins, delphinidin) were also found in various combinations across the Gesnerioideae (Figure 3 and Supplementary Figure S1). A previous study on Solanaceae reported that, even though the species of this family were able to produce all three main groups of anthocyanins, they often produce only one type of anthocyanin or a mix of two anthocyanins with consecutive hydroxylation levels (e.g., pelargonidin-cyanidin or cyanidin-malvidin), and none of the studied species produced all three pigment types (Ng et al., 2018). It has been proposed that the anthocyanin composition is constrained by the stepwise structure of the biosynthetic pathway that leads to the successive production of the red mono-hydroxylated pelargonidin, purple di-hydroxylated cyanidin, and the blue tri-hydroxylated delphinidin pigments (des Marais and Rausher, 2008; Ng et al., 2018). In the Gesnerioideae, anthocyanin composition is less constrained. Although we observed combination of pigments with the same or consecutive hydroxylation levels more often than other combinations (Supplementary Figure S1), we also observed pigments with non-consecutive hydroxylation levels together and frequently found up to five pigment types in the same species. This ability to utilize different anthocyanin combinations from two different branches makes anthocyanin-based floral color a highly flexible trait in the Gesnerioideae.

### The Effect of Anthocyanin Composition on Floral Color Diversity

Repeated evolution of orange/red flowers occurred frequently in angiosperms (Cronk and Ojeda, 2008; Ng et al., 2015, 2016). Biochemical changes associated with these floral color transitions mainly involve an increased production of red mono-hydroxylated pelargonidin pigments (des Marais and Rausher, 2008), carotenoids (Tanaka et al., 2008), or a combination of both (Ng and Smith, 2016). In Gesnerioideae, orange/red flowers have evolved several times in different lineages, such as in the genera *Columnea* (Schulte et al., 2015), *Drymonia* (Clark et al., 2015), *Nematanthus* (Serrano-Serrano et al., 2015), and *Sinningia* (Perret et al., 2007; Figure 4). We found that the convergent evolution of the red color does not rely on the same biochemical basis but on the activation of different branches of the ABP, resulting in the production of up to five different pigments in a single flower. Deoxyanthocyanins and the pelargonidin derivatives contribute to the majority (83%) of the orange/red flowers. Beside these major orange/red pigments, we also found di-hydroxylated cyanidin and tri-hydroxylated malvidin, either alone or in combination with other compounds, in some other orange/red flowers like in *Vanhouttea hilariana*. Although these pigments are frequently associated with pink and purple/blue flowers, it has been shown that these anthocyanins can produce red under low vacuolar pH conditions (Yoshida et al., 2003; Tanaka et al., 2008). These results demonstrate that Gesnerioideae evolved several different biochemical solutions to produce orange/red flowers, which indicates that convergent evolution of orange/red color does not result from parallel changes at the biochemical level but instead produced via the activation of different branches of the ABP.

Beside anthocyanins, other pigment types may also be involved in the floral color in the Gesnerioideae. For example, the pale green flowers of *Rhytidophyllum exsertum* and *Sinningia brasiliensis* have chloroplast in their parenchyma, and the overlay of cyanidin-producing epidermal cells result in the brown dots typical of these bat-pollinated flowers (Sanmartin-Gajardo and Sazima, 2005; Martén-Rodríguez et al., 2015). Carotenoids may also contribute to the yellow floral colors in the Gesnerioideae samples where no anthocyanidins were detected. To date, carotenoids have been recorded in the genus *Achimenes*, *Columnea* (*C. crassifolia, C. linearis, C. rutilans*, and *C. woodii*) and in *Titanotrichum oldhamii* (Harborne, 1967; Roberts and Roalson, 2017), but the overall presence of carotenoids in the Gesneriaceae family has not been studied in detail.

### The Effect of Floral Anthocyanin Type on Color Discrimination of Pollinators

Flower color has a direct impact on their visual discrimination by pollinators. A fit between visual systems and specific color signals has been observed in communities with bees and birds as pollinators (Burd et al., 2014; Papiorek et al., 2016; Shrestha et al., 2016; Camargo et al., 2019). However, little is known about how these color signals evolved to match the visual systems of their principal pollinators during plant radiations. Using both analysis of spectral marker points (Figure 6) and color space models for bees and birds (Figure 5), we show that the extent of floral color diversity in Gesnerioideae depends on which ABP branch is activated. Species relying on the hydroxyanthocyanin branch of the ABP pathway display a wide range of color and marker points, and they match the visual capacities of both bees and hummingbirds. The visual color space occupied by deoxyanthocyanin-producing flowers is smaller than the hydroxyanthocyanin-producing flowers, and these flower colors better match the visual capacities of hummingbirds. The effect of these floral spectral specificity on pollinator preferences is confirmed by pollinator observation data, which indicate that hydroxyanthocyanin-producing species are either pollinated by bees or hummingbirds, whereas flowers with deoxyanthocyanin are only pollinated by hummingbirds. The lack of bee-pollination within the deoxyanthocyanin-producing flowers indicate that this type of floral pigment is particularly efficient at limiting bee visitation by constraining flower reflectance in the long-wavelength ranges that are better detected by hummingbirds than bees (Lunau et al., 2011; Bergamo et al., 2016).

Even though the shift from the hydroxy- to deoxy-anthocyanin branch may decrease the subsequent phenotypic and ecological diversification of a clade, relying on deoxyanthocyanins as floral pigments might still be evolutionary beneficial. Indeed, constraining the deoxyanthocyanin-producing clades to rely only on hummingbird pollination maybe advantageous, because this pollinator group is particularly efficient in the rainforests of mountain systems with a high Gesnerioideae diversity (Perret et al., 2013; González et al., 2015). In addition, transitions to hummingbird pollination has been shown to promote diversification not only in the Gesnerioideae (Roalson and Roberts, 2016; Serrano-Serrano et al., 2017), but also in many other Neotropical clades (Kay et al., 2005; Wilson et al., 2006; Schmidt-Lebuhn et al., 2007; Tripp and Manos, 2008).

## Conclusion

We have shown that the Gesnerioideae flowers show remarkable color diversity, which is due to the use of the ABP up to its full capacity, including the deoxyanthocyanin branch, which is rarely employed in angiosperms. Utilizing both branches in the ABP provides Gesnerioideae with alternative options for floral color production without forcing an evolutionary dead-end, resulting in extraordinary floral color diversity and different signaling strategies adapted for bees and hummingbirds. By improving our understanding the biochemical basis of floral color in the Gesnerioideae, we provide clear perspectives to identify the genetic changes responsible for floral color shifts and test the effects of these transitions on plant-pollinator interactions and angiosperm diversification.

## Data Availability Statement

The original contributions presented in the study are included in the article/Supplementary Materials, further inquiries can be directed to the corresponding author/s.

## Author Contributions

EO contributed to data collection, data analysis, and manuscript writing. KD contributed to data collection and data analysis. MW, LC, and CG contributed to data collection and manuscript review. GG contributed to data analysis and manuscript review. MP contributed to supervision and manuscript writing. All authors contributed to the article and approved the submitted version.

## Conflict of Interest

The authors declare that the research was conducted in the absence of any commercial or financial relationships that could be construed as a potential conflict of interest.

## Footnotes

1 http://dnp.chemnetbase.com
