# Supplementary figures and images for: Chemical Basis of Floral Color Signals in Gesneriaceae: The Effect of Alternative Anthocyanin Pathways
Source: Front Plant Sci. 2020 Dec 14;11:604389. doi: 10.3389/fpls.2020.604389 (PMC7767864; doi:10.3389/fpls.2020.604389)

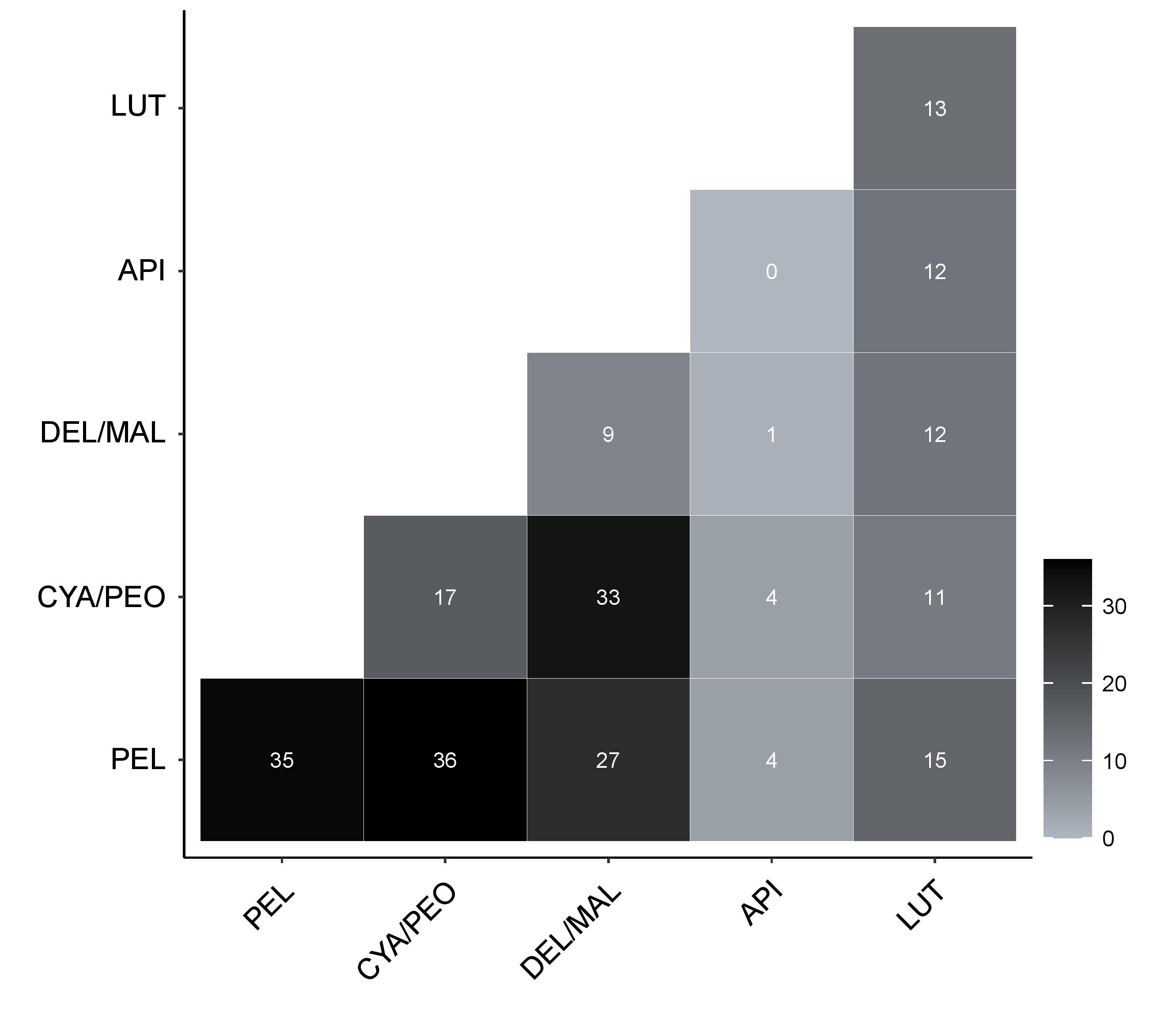

Supplement: Supplementary file 1 [file Image_1.JPEG]

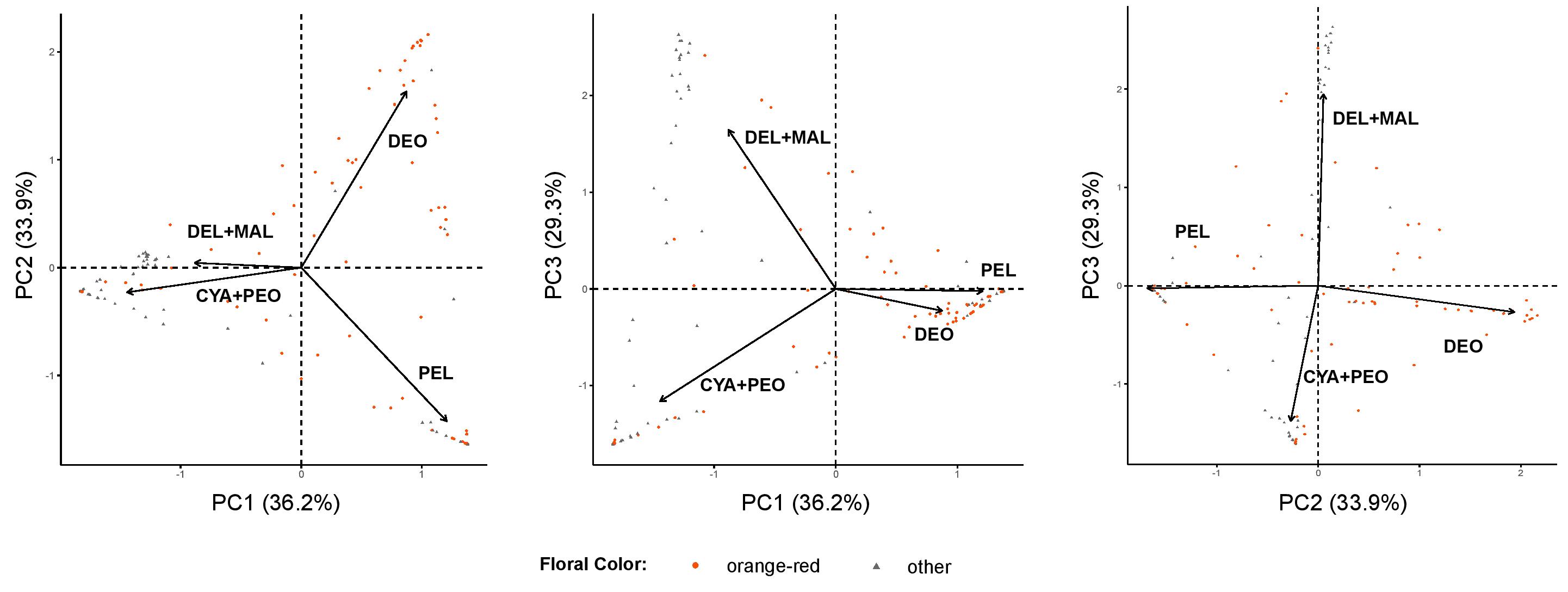

Supplement: Supplementary file 2 [file Image_2.jpg]
